# Is asciminib an effective tyrosine kinase inhibitor for chronic myeloid leukemia patients with tyrosine kinase inhibitor resistance?

**DOI:** 10.1016/j.htct.2026.106257

**Published:** 2026-02-22

**Authors:** Musab MA Omar, Majed A Alanazi, Denise E. Jackson

**Affiliations:** a Thrombosis and Vascular Diseases Laboratory, School of Health and Biomedical, Sciences, STEM College, RMIT University, PO Box 71, Bundoora, Melbourne, VIC 3083, Australia; b Department of Clinical Laboratory Sciences, College of Applied Medical Sciences, Taibah University, Madinah 42353, Saudi Arabia; c Ministry of Education, Riyadh, Saudi Arabia

**Keywords:** Chronic myeloid leukemia, Tyrosine kinase inhibitors, Asciminib, Drug resistance, Combination therapies

## Abstract

Asciminib represents a significant advancement in the treatment of chronic myeloid leukemia, establishing a novel therapeutic paradigm by specifically targeting the ABL1 myristoyl pocket, a mechanism distinct from that of conventional adenosine triphosphate-competitive inhibitors. Such a selective inhibitor offers an alternative treatment strategy for patients with chronic myeloid leukemia who have developed resistance to previous tyrosine kinase inhibitor therapies. Although asciminib demonstrates a superior safety profile, primarily characterized by a reduction in cardiovascular adverse events associated with prior tyrosine kinase inhibitors, its clinical significance extends further. The effectiveness of asciminib, combined with its capacity to overcome resistance through combination strategies with adenosine triphosphate-binding site tyrosine kinase inhibitors, establishes it as a focal point in emerging chronic myeloid leukemia treatment approaches. It remains essential to continue research and clinical trials to enhance the therapeutic efficacy of asciminib and manage its associated side effects.

## Overview of chronic myeloid leukemia

**Figure 1:**
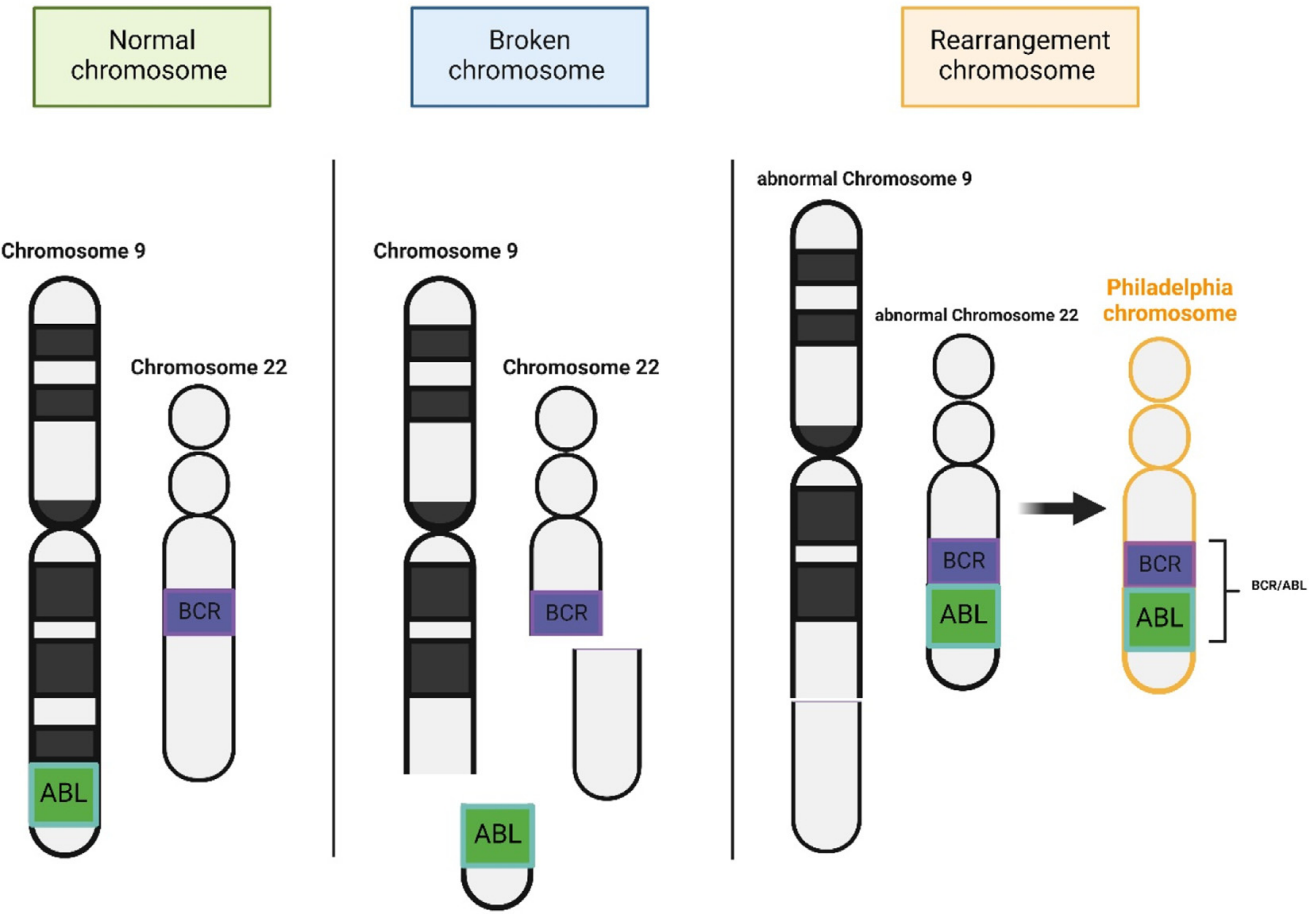
Schematic representation of the t(9;22)(q34;q11) translocation, which facilitates the formation of the Philadelphia chromosome. The ABL1 gene is located on the long arm of chromosome 9, while the BCR gene is situated on chromosome 22. This reciprocal translocation results in the formation of the BCR::ABL1 fusion gene on the derivative chromosome 22, commonly referred to as the Philadelphia chromosome.

Chronic Myeloid Leukemia (CML) is a myeloproliferative neoplasm genetically defined by the presence of the Philadelphia chromosome (pH). This chromosome results from a reciprocal translocation between chromosomes 9 and 22, t(9;22)(q34;q11), wherein the ABL1 gene from chromosome 9 is fused with the BCR gene on chromosome 22, leading to the formation of the BCR::ABL1 fusion gene (Figure 1) [1]. The BCR::ABL1 fusion gene is responsible for the production of constitutively active BCR::ABL1 tyrosine kinase which is considered to be the core of CML pathogenesis due to its function of activating essential signaling pathways including the PI3K/AKT, RAS/RAF/MEK/ERK, and JAK/STAT pathways [1]. These pathways contribute to uncontrolled cell growth, proliferation, and survival, underlying the disease's escalating nature. The fusion of BCR and ABL genes disrupts the normal regulation of ABL1 kinase activity, leading to the malignant transformation of myeloid cells [1].

CML represents approximately 15 % of all leukemia cases, predominantly affecting adults with a median age of 55 years at diagnosis [2]. It progresses through three phases: chronic, accelerated, and blast crisis, with most diagnoses occurring in the chronic phase [2]. Initially, the disease is characterized by a bone marrow expansion of myeloid cells, causing fatigue, splenomegaly, and anemia. If left untreated, CML may progress to more aggressive phases, culminating in a blast crisis characterized by increased blast cells and genetic instability, often resulting in death [2].

Tyrosine kinase inhibitors (TKIs), such as imatinib, nilotinib, and dasatinib, have revolutionized CML treatment, greatly improving survival rates. These drugs specifically inhibit the BCR::ABL1 tyrosine kinase, leading to remission in the majority of patients. However, further research for new therapeutic targets and strategies is essential because long-term use has revealed issues with drug resistance [3].

The treatment of CML has evolved over recent decades. Initially, physicians would conduct observations of the patient’s condition, the symptomatic presentation of the disease and how it progressed as well as alternative treatment paths involving combining chemotherapy with medication [4]. Stem cell transplants are another treatment path. Some circumstances necessitate the administration of interferon-alpha (IFNα) and TKIs. Frequently, practitioners combine therapies to enhance the effectiveness of treatment approaches. As there is no cure for CML to date, these approaches help to manage the disease [4].

Predicting treatment outcomes remains challenging, despite the availability of highly efficacious therapeutic options. Current predictive models do not fully account for the long-term response of individual molecular markers to treatment. Furthermore, there is a lack of comprehensive data regarding the kinetic profiles of these markers or the associated risk of molecular relapse for individual patients. Even the kinetics of BCR::ABL1 transcripts, which provide insights into molecular responses, are limited by the fact that samples are typically derived from peripheral blood. Consequently, these data may not accurately reflect the levels of minimal residual disease within the bone marrow. This discrepancy is primarily attributable to the difficulty of directly accessing and monitoring the leukemic stem cell compartment within the bone marrow environment [5].

A major advancement in the management of CML was the development of first-generation TKIs such as imatinib, which achieve high rates of sustained cytogenetic responses. [6] A significant challenge, however, is resistance [7]. Second-generation tyrosine kinase inhibitors were developed to overcome imatinib resistance and intolerance. An example is nilotinib, which exhibits significantly higher potency against the BCR::ABL1 oncoprotein and possesses a distinct profile of adverse events compared to first-generation therapies [8]. The composition of nilotinib is similar to that of imatinib, but modifications were made to enhance activity and selectivity improve its efficacy against most imatinib-resistant BCR::ABL1 mutations, except for the T315I mutation [9,10].

The recognition of the role of T315I mutation in resistance led to the development of third-generation TKIs like ponatinib and asciminib. Ponatinib was the first third-generation TKI designed to overcome resistance attributable to the T315I mutation through its inhibitory action facilitated by the creation of an ethynyl bond [11]. Ponatinib incorporates a carbon-carbon triple bond (ethynyl linker) that allows the molecule to bypass the steric hindrance caused by the T315I mutation [11].

## Asciminib in chronic myeloid leukemia management

**Figure 2:**
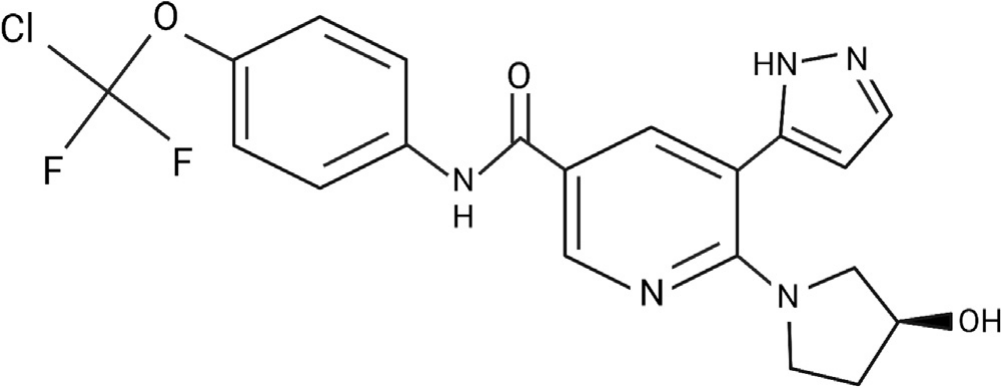
Chemical structure of asciminib, a first-in-class Specifically Targeting the ABL Myristoyl Pocket inhibitor. Structurally, asciminib features a dichlorodifluoromethoxyphenyl moiety coupled to an amide-linked central phenyl ring. This central scaffold is conjugated to a pyrimidine ring, which is further substituted with a piperidine ring bearing a terminal hydroxy group. Unlike traditional ATP-competitive TKIs, asciminib functions through allosteric inhibition by mimicking the regulatory myristoylated N-terminus of ABL1. This restores the autoinhibitory conformation of the kinase, a mechanism typically lost following the BCR::ABL1 oncogenic fusion, thereby providing a potent therapeutic alternative for patients with acquired TKI resistance [13].

Asciminib is still considered an investigational drug. However, it is gaining popularity because of its capacity to specifically inhibit the tyrosine kinase activity of native ABL1 and that of the chimeric BCR::ABL1 oncoprotein responsible for CML [12]. It was granted breakthrough therapy designation by the U.S. Food and Drug Administration (FDA) on 9 February 2021 to treat patients with chronic phase Philadelphia chromosome positive chronic myeloid leukemia (pH^+^ CP-CML) who had been treated with two or more TKIs and pH^+^ CP-CML patients with the T315I mutation [12]. Figure 2 shows the chemical structure of asciminib.

**Figure 3:**
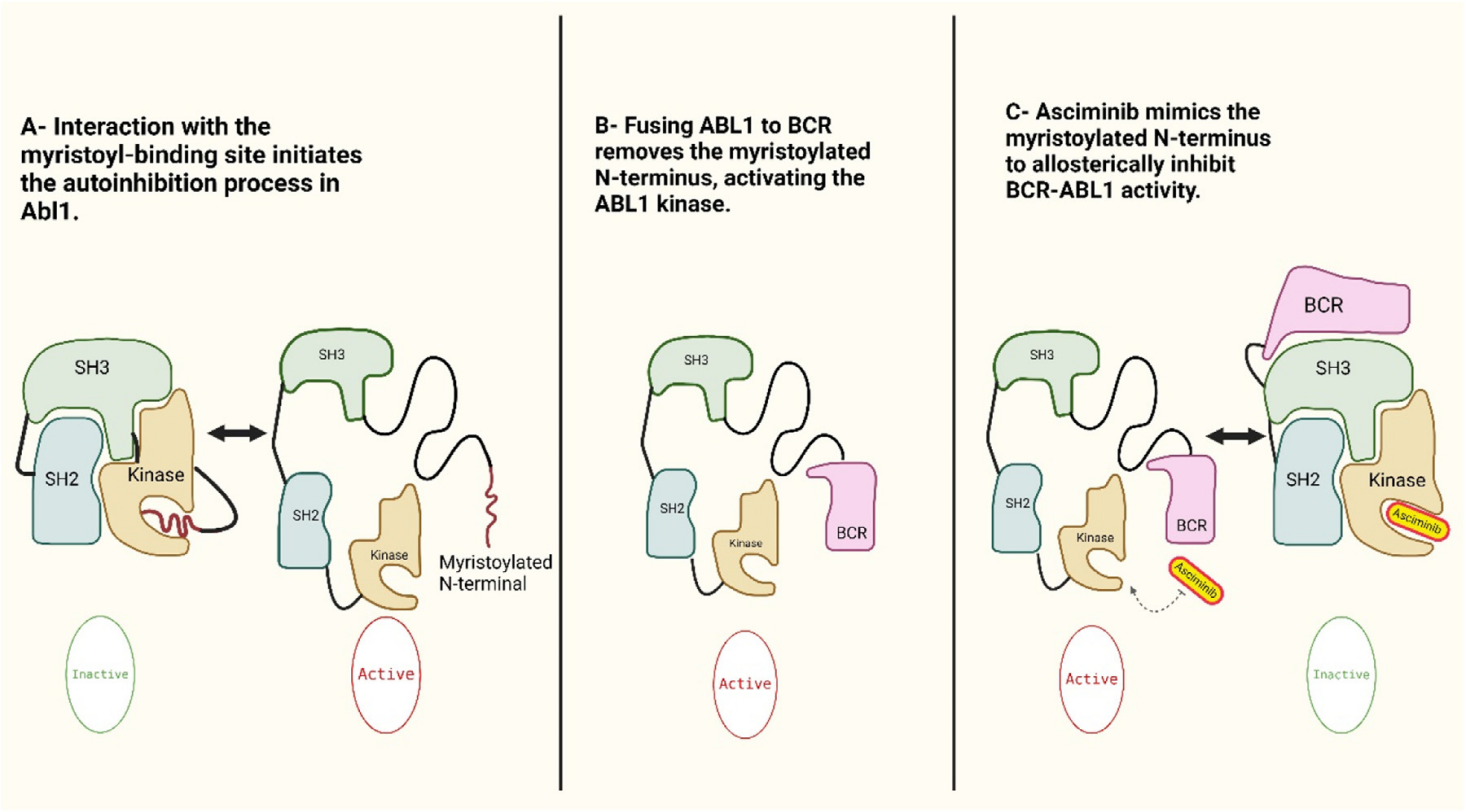
Asciminib binds specifically to the allosteric myristoyl pocket of the BCR::ABL1 oncoprotein. Under physiological conditions, autoinhibition of the ABL1 kinase is achieved through the binding of the myristoylated N-terminus to the myristoyl-binding site (A). This regulatory motif locks the ABL1 kinase in its inactive conformation (B). However, the oncogenic fusion of BCR and ABL1 results in the loss of the myristoylated N-terminus, leading to constitutive kinase activation (B). Asciminib mimics the natural myristoyl ligand by binding to this allosteric site, thereby restoring the autoinhibited state and inhibiting BCR::ABL1 kinase activity (C) [15].

Unlike tyrosine kinase inhibitors that target adenosine triphosphate (ATP)-binding sites, asciminib, a Specifically Targeting the Abelson Myristoyl Pocket (STAMP) inhibitor, binds to the myristoyl pocket of the Abelson tyrosine kinase 1 protein. This agent was granted accelerated approval in the treatment of CML by the FDA in October 2021 [14]. The pharmacological action of the drug impedes the interaction between the myristoyl group and the myristate pocket of the ABL1 protein [12]. This obstruction precludes the regulatory function of the myristoyl group upon its conjugation with BCR, thereby inhibiting subsequent enzymatic activity [12]. Figure 3 shows the mode of action of asciminib.

No clinically significant adverse outcomes have been reported regarding its off-target activity. This favorable safety profile is likely attributable to the high specificity of the inhibitor toward cells expressing the BCR::ABL1 oncoprotein, alongside its negligible impact on non-kinase targets in comprehensive biochemical screens [16]. Without binding to the ATP-binding site, asciminib is mainly focused on inhibiting kinase domain mutations that lead to the drug resistance seen in earlier generations of ATP-competitive drugs [16]. Binding to the ABL1 myristoyl binding pocket is vital to enable asciminib to inhibit BCR::ABL1 in a non-ATP competitive way. The drug has an almost non-overlapping point mutation-driven resistance profile which is superior to ATP-competitive TKIs [17]. Nevertheless, studies have identified specific BCR::ABL1 mutations that diminish the anti-proliferative activity of the drug, with certain alterations leading to clinical resistance. Despite these challenges, asciminib maintains high potency against the majority of known BCR::ABL1 mutations, offering a robust therapeutic option for patients who have developed resistance to other inhibitors [17]. However, this does not guarantee that it will always be like that. There are emerging mutations, including V468F, I502L, P465S, and A337V/T, for which the drug is ineffective. These mutations are localized within the myristoyl-binding pocket. Consequently, they confer resistance to asciminib, rendering the treatment [17]. As a result of resistance to mutations, transformations from the accelerated phase to the blast phase are recorded in 4.6 % and 7.3 % of patients on dasatinib and imatinib, respectively [18].

Asciminib may suit patients with significant vascular risk factors or a history of cardiovascular diseases, as well as patients with documented toxicities thought to be related to the class of ATP-competitive TKIs including nilotinib and ponatinib [14]. The drug may also be the choice for patients who do not achieve time-dependent molecular targets but are TKI-responsive [14]. Patients are prone to adverse events due to intolerance and resistance that often lead to dose interruptions or reductions. The current data suggest that the safety profile of asciminib is relatively favorable but the view is expected to keep evolving as long-term data become available to reflect the true incidence of toxicities [14].

## Mechanism of action of asciminib

Asciminib serves as a selective allosteric inhibitor of BCR::ABL1 by mimicking the natural autoinhibitory mechanism of ABL1. In the native ABL1 protein, the N-terminal myristoyl group binds to a specific hydrophobic pocket to maintain the kinase in an inactive state. However, this autoinhibitory capacity is lost upon the formation of the BCR::ABL1 fusion protein [14]. The binding of asciminib mimics the natural interactions within the myristoyl pocket, thereby inhibiting BCR::ABL1 and restoring allosteric control over its kinase activity. This mechanism establishes asciminib as a first-in-class inhibitor targeting the ABL1 myristoyl pocket. The molecule was developed through the structural optimization of GNF-2 and GNF-5; the former served as a pivotal tool compound in validating the allosteric inhibition of BCR::ABL1 and demonstrating that targeting this site effectively suppresses kinase activity. This proof-of-concept was fundamental to the subsequent development of asciminib. Although asciminib shares the non-ATP-competitive biochemical kinetics characterized by GNF-2, it represents a significant clinical advancement, exhibiting approximately 100-fold greater potency [17].

The myristoylic domain physiologically serves as a negative control for kinase activity. Its function is lost in CML. The limited availability of the myristoyl pocket in other kinases means that the molecule is highly selective for BCR::ABL1. The highly selective nature of asciminib for ATP site mutants such as T315I and unmutated BCR::ABL1 is inhibited by the ABL1 and ABL2 kinases [14]. Asciminib helps to recover the original autoregulatory mechanism that induces the inactive conformation and inhibits downstream signaling [14].

## Comparing asciminib with other tyrosine kinase inhibitors

Asciminib demonstrates a favorable safety profile alongside statistically significant and clinically meaningful superior efficacy compared to ATP-competitive inhibitors such as bosutinib [19]. These findings support its utilization as a preferred therapeutic strategy for CML, particularly for patients who have failed to respond to conventional ATP-binding site inhibitors. The unique mechanism of action differentiates it from ATP-competitive TKIs. First, it works by specifically targeting the ABL myristoyl pocket. Furthermore, ABL1 under normal conditions is regulated by the binding between the myristoylated N-terminal and the myristoyl-binding pocket, which renders the kinase inactive. Another factor that differentiates asciminib is the loss of autoregulation in CML due to the formation of the constitutively active BCR::ABL1 fusion oncoprotein. Binding to the myristoyl-binding pocket restores the inactive conformation leading to the inhibition of ABL1 kinase [16,17].

## Adverse events of asciminib

It has been reported that asciminib can cause a variety of adverse events, including pancreatitis and elevated blood pancreatic enzyme levels, both of which are serious, but relatively uncommon. It is necessary to monitor blood counts regularly due to myelosuppression, which can manifest as thrombocytopenia, neutropenia, and anemia [20]. In the ASCEMBL trial, Grade ≥3 neutropenia occurred in approximately 32 % of patients, thrombocytopenia in 24 %, and anemia in 5 % [15]. According to the Australian Scemblix product information, Grade 3 or higher adverse events included thrombocytopenia (18.5 %), neutropenia (15.7 %), elevated pancreatic enzymes (12.4 %), hypertension (8.4 %), and anemia (5.3 %). The most recent FDA Prescribing Information (2024) reported higher incidences of Grade 3 or higher neutropenia (13 %), thrombocytopenia (17 %), anemia (4 %), and elevated pancreatic enzymes (9 %), based on large pooled data sets. These data confirm the reproducible hematologic profile of asciminib while indicating a lower incidence of severe off-target toxicity compared to ATP-binding TKIs. In the presence of elevated liver enzymes, liver function abnormalities suggest potential hepatotoxicity and should be monitored closely [8]. As well as fatigue, patients have reported musculoskeletal pain as well as gastrointestinal disturbances like diarrhea and dermatological reactions such as rashes [20]. It shows that the adverse cardiovascular events of asciminib are strikingly different to ATP-pocket TKIs, such as ponatinib and nilotinib, offering therapeutic advantage to patents with compounded risk of cardiovascular disease.

## Management of adverse events

To advance CML management by decreasing or even eliminating the adverse events of asciminib requires a dynamic and integrative process [15]. Patients with pancreatitis and elevated pancreatic enzymes require close monitoring for abdominal signs and symptoms; accordingly, asciminib therapy may need to be interrupted or discontinued based on the severity of the condition [20]. Monthly full blood counts are needed to investigate myelosuppression with dose adjustment or temporary discontinuation of therapy being required if a cytopenia is identified [20]. For early detection of liver toxicity, biochemistry profile tests including liver function tests should be conducted on a regular basis, and the dose of asciminib should be adjusted or the drug discontinued as possible interventions [21]. Medications such as analgesics can be used as part of musculoskeletal pain management. Moreover, rehabilitation exercises can often be useful to improve physical function. In the case of gastrointestinal side effects, such as diarrhea, dietary changes, hydration, and antidiarrheal medications can all be helpful. A topical treatment may be used to address dermatological reactions, whereas systemic therapies may be used if the reaction is severe [22]. In order to take action early, patients must be educated to recognize symptoms and report them in a timely manner. Finally, the treatment plan should consider a cardiovascular risk assessment, given the favorable profile of asciminib [17].

## Comparison with other chronic myeloid leukemia treatments

When compared to other treatments for CML, asciminib represents a significant advancement [17]. Unlike imatinib, a first-generation TKI notorious for gastrointestinal reactions, fluid retention, cramps, and rash, asciminib has other adverse events, such as myelosuppression and liver enzyme elevations [11], [17].

The combination of asciminib with second-generation TKIs such as nilotinib and dasatinib is safer than second-generation TKIs alone, which can result in serious cardiovascular toxicities like QT prolongation, arterial occlusive disorders, pleural effusions, and pulmonary hypertension [21]. Patients at risk of or with existing cardiovascular diseases find its lower rate of cardiovascular adverse events particularly attractive [17,21].

Asciminib is also safer than the third-generation TKI ponatinib, which is associated with an elevated risk of arterial thrombotic events [17]. In patients with cardiovascular risk factors or a history of vascular complications, asciminib is likely a better choice than ponatinib since it requires less cardiovascular monitoring [17].

## Resistance to asciminib in chronic myeloid leukemia treatment

The primary cause of BCR::ABL1 resistance to asciminib is the emergence of specific genetic mutations within the kinase domain. This underscores the mechanistic distinction between asciminib and conventional ATP-competitive TKIs; specifically, asciminib binds to the myristoyl pocket, representing a novel allosteric approach to inhibiting the BCR::ABL1 oncoprotein. While mutations such as T315I confer resistance to many ATP-site inhibitors, they do not typically affect the myristoyl-binding site. However, novel missense mutations within the C-terminal lobe can disrupt the architecture of the pocket, thereby preventing drug binding and leading to clinical relapse [17]. Nevertheless, mutations that affect the structure and orientation of the myristoyl pocket are responsible for loss of affinity with asciminib which consequently causes resistance. Furthermore, resistance can also be caused by BCR::ABL1 independent mechanisms, for example, altered efflux transport or alternative signaling pathway activation which mediate resistance.

## Detection and monitoring of resistance

The most frequently applied genetics and molecular methods used to recognize and track asciminib resistance are Next Generation Sequencing (NGS) and Quantitative Polymerase Chain Reaction (qPCR). These advanced techniques detect possible mechanisms of resistance due to mutations within the BCR::ABL1 gene. By routinely tracking BCR::ABL1 transcripts, early resistance to treatment can be recognized and the effectiveness of treatment assessed.

## Impact on treatment strategy

The emergence of resistance to asciminib significantly impacts CML therapeutic strategies. To address resistance mediated by specific mutations, combination therapies targeting multiple signaling pathways or employing diverse mechanisms of action may be required for patients with advanced-stage CML. Utilizing agents that target downstream signaling or combining asciminib with other TKIs represents a viable approach to overcoming such resistance [23]. Furthermore, upon the detection of resistance, treatment must be tailored to the patient’s specific mutational profile. Potential interventions include switching to an alternative TKI, dose optimization, or considering allogeneic stem cell transplantation in advanced disease states [23]. Ultimately, prioritizing the understanding and management of asciminib resistance through ongoing research and innovative strategies is essential to optimize clinical outcomes.

## Combination strategies with asciminib

Asciminib demonstrates a favorable safety profile as well as statistically significant and clinically meaningful superior efficacy compared to ATP-competitive inhibitors such as bosutinib [19]. These findings support the use of asciminib as the preferred therapeutic option, particularly for patients who have demonstrated resistance or intolerance to previous TKIs. Its unique mechanism of action, characterized by specifically targeting the ABL myristoyl pocket, distinguishes it from conventional ATP-competitive TKIs. Under physiological conditions, ABL1 activity is regulated by the binding of its myristoylated N-terminus to the myristoyl-binding pocket, which maintains the kinase in an autoinhibited, inactive state. Asciminib restores this native autoinhibitory function by mimicking the binding of the myristoyl group, a regulatory mechanism that is otherwise lost following the oncogenic fusion of BCR and ABL1 [16,17].

## Rationale for combination therapy

The main reasons combination treatment with asciminib is chosen to treat CML patients are to increase treatment efficacy, overcome treatment resistance, and decrease adverse events. Asciminib targets the ABL myristoyl pocket by inhibition and can be combined with different TKIs which target different pockets or stages in the proliferation of cancer cells. The rationale behind this strategy is to provide a more comprehensive blockade of BCR::ABL1 oncogenic signaling with this strategy potentially leading to a deeper molecular response, and fewer resistance mechanisms as well as better flexibility in terms of addressing different patient requirements and disease characteristics [23].

## Current research on combination therapies

The combination using asciminib along with an ATP-pocket inhibitor such as imatinib, nilotinib, or ponatinib is a key interest of ongoing research. Initial results indicate that this combination is beneficial for single-agent TKI resistance such as imatinib resistance as well as in CML patients with complex pathophysiology [23].

**Figure 4:**
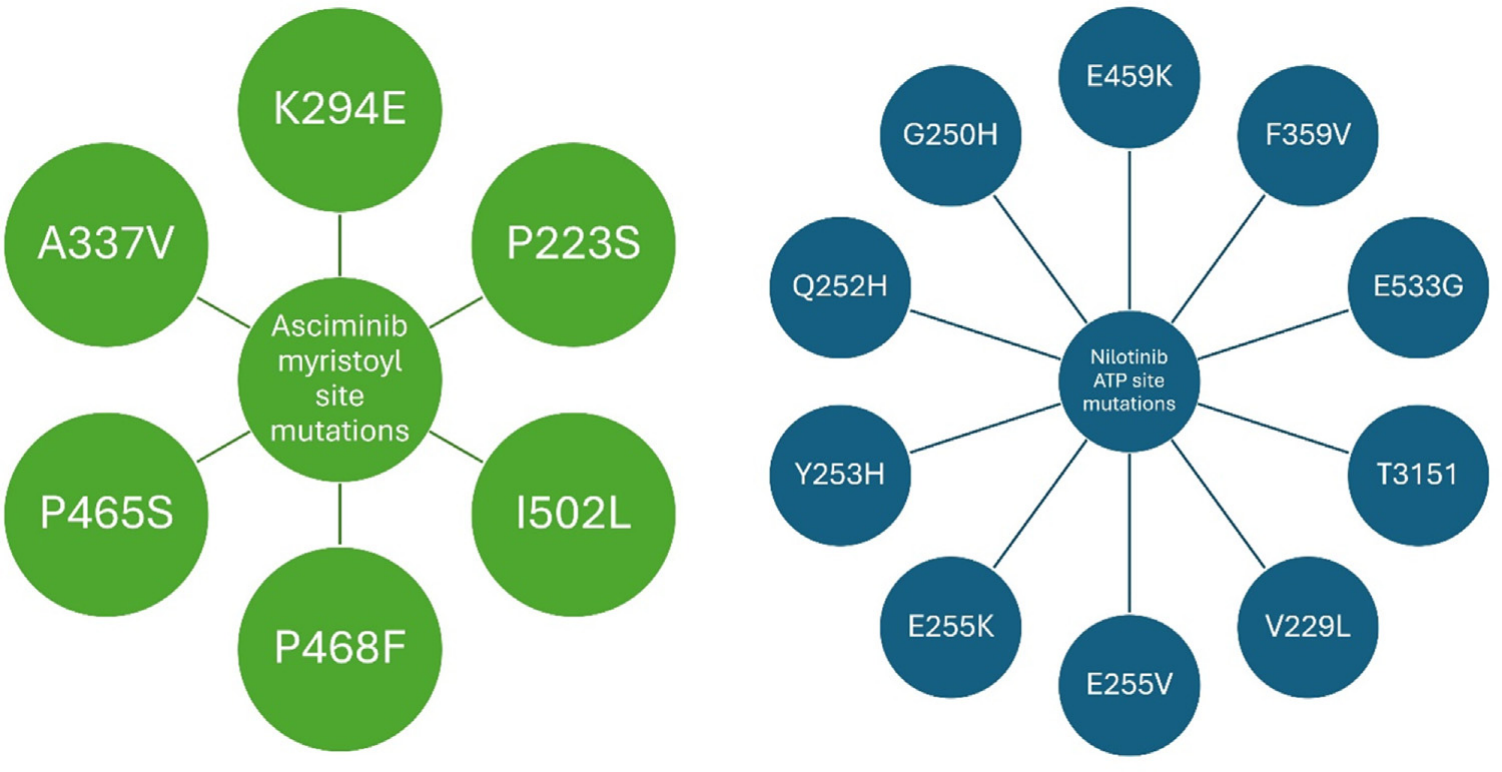
Resistance mutations for asciminib and nilotinib. Green circles denote mutations within the myristoyl pocket that confer resistance to asciminib. These are distinct from the blue circles on the right, which represent ATP-binding site mutations conferring resistance to nilotinib. Because these mutations occur in spatially separate domains of the BCR::ABL1 protein, they highlight the lack of cross-resistance between allosteric and ATP-competitive inhibitors, providing a mechanistic rationale for combination therapy.

The use of asciminib along with other TKIs significantly inhibits the expression of resistance point mutations in Ba/F3 [17]. Ba/F3 as laboratory-grown cells, are a useful tool in experimental combination trials. It is possible to genetically manipulate these cells to have particular mutations of the BCR::ABL1 gene. By testing Ba/F3 cells it is possible to evaluate the efficacy of asciminib to target various BCR::ABL1 mutations. This enables an understanding of how effective combinations of drugs will be in patients with certain genomic characteristics of leukemia especially in respect to drug resistance [17]. Combination strategies show more effective management in CML patients than the use of only one TKI. Even if a compound mutation confers resistance to either drug separately, combining asciminib with ponatinib is effective against Ba/F3 cells that harbor BCR::ABL1 compound mutations at therapeutically-relevant concentrations [17]. Apart from prolonging survival, the combination inhibits hematological malignancy in a T315I-inclusive compound mutant model. The complementary resistance profiles of asciminib and nilotinib facilitate the complete regression of hematologic malignancies when administered in combination. In contrast, resistance mediated by specific point mutations persists when either agent is utilized as a monotherapy [17]. Mutations within the myristoyl and ATP-binding sites can collectively confer resistance to both asciminib and nilotinib. Emerging evidence suggests a significant overlap between the specific residues involved in ATP-binding site resistance and those that impact the efficacy of established ATP-competitive TKIs [17]. Figure 4 illustrates the mutational landscape of resistance to asciminib and nilotinib; notably, the myristoyl pocket mutations associated with asciminib resistance are spatially and mechanistically distinct from the ATP-binding site mutations that confer resistance to nilotinib [17].

As García-Gutiérrez et al. noted, the mechanism of action that targets the myristoyl pocket, when combined with TKIs targeting the ATPase domain of BCR::ABL1, helps to suppress the emergence of resistance [24]. However, dose escalation with ponatinib is not a feasible option because of dose-limiting toxicities [17]. Combining asciminib with ponatinib allows for lower doses of ponatinib to target highly-resistant BCR::ABL1 compound mutations [17].

## Efficacy and safety of combination approaches

Early clinical trials demonstrated that combination therapies involving asciminib can achieve deeper and more durable responses in CML [23]. However, it is essential to note that combination therapies may exacerbate existing adverse events or induce novel toxicities in CML patients. Future research aimed at optimizing CML management through combination strategies must carefully balance therapeutic efficacy with the safety profile of the treatment [17].

## Clinical trials of asciminib as a monotherapy

Asciminib monotherapy is frequently indicated for patients who have exhausted conventional therapeutic options or those harboring the T315I gatekeeper mutation. Its unique mechanism of action (specifically targeting the ABL1 myristoyl pocket) enables continued efficacy in cases where traditional ATP-competitive TKIs fail due to binding-site alterations [16]. For instance, a Phase 1 study of asciminib as monotherapy was used to demonstrate significant efficacy in patients suffering from CML who had shown resistance to at least two TKIs. In the same study, a major molecular response was observed in 48 % of patients, and 57 % of patients achieved complete cytogenetic response (CCyR) at 24 weeks of treatment [25]. Similarly, the hematological remission rate, used to determine the normalization of blood counts in another study, was achieved in 95 % of the patients. Two ongoing trials (ASC4START and ASC4FIRST) further investigate the efficacy of monotherapy as a first-line treatment for patients newly diagnosed with CML in the chronic phase [26]. Therefore, the studies above reveal the efficacy of asciminib as a monotherapy in patients with heavily pretreated CML. Thus, it provides a promising alternative, especially for patients who have exhausted other treatment options.

## Clinical trial in combination with adenosine triphosphate-pocket inhibitors

In instances where monotherapy proves insufficient due to emergent resistance or suboptimal molecular response, combining asciminib with ATP-competitive TKIs may be necessary. This dual-targeting approach represents a promising strategy to circumvent TKI resistance and achieve deeper molecular responses in CML patients [27]. For instance, asciminib was evaluated in combination with ATP-pocket inhibitors in a Phase 3 clinical trial. It yielded improved outcomes compared to using ATP-pocket inhibitors as a monotherapy. In one such study, 72 % of patients receiving asciminib in combination with imatinib achieved a major molecular response at 24 weeks, compared to 51 % of patients in the control group receiving imatinib monotherapy. In yet another study, combination therapy yielded a better response, with 65 % of patients achieving a deep molecular response, defined as a BCR::ABL1 transcript level of ≤0.01 %, compared to 42 % in the control group [17].

## Conclusion

Asciminib, characterized as an allosteric myristoyl pocket inhibitor rather than a conventional ATP-competitive inhibitor, offers superior management compared to earlier CML therapies. A primary advantage of asciminib is its ability to circumvent the resistance and adverse events associated with traditional TKIs, particularly in patients with advanced-stage CML. The development of asciminib addressed a critical clinical need for advanced management strategies that surpass the efficacy of previous approaches to improve patient outcomes and quality of life. Asciminib serves as a vital alternative for patients experiencing relapse, intolerance, or resistance to prior TKIs. Furthermore, the strategic combination of asciminib with other TKIs represents a promising approach to mitigating adverse effects and enhancing therapeutic depth. The preliminary success of these combination regimens has solidified the role of asciminib in the evolving landscape of CML treatment.

The future role of asciminib in CML management remains contingent upon the outcomes of extensive longitudinal research. It is imperative to conduct long-term clinical trials to comprehensively evaluate the efficacy and safety profile of asciminib in both monotherapy and combination settings. Such research is critical to identify optimal therapeutic protocols, elucidating emergent resistance mechanisms, and devising strategies to mitigate them. Furthermore, exploring synergistic interactions between asciminib and novel therapeutic modalities will be pivotal to maximizing the clinical potential of STAMP inhibition.

## Data availability

The data that support the findings of this study are available from the corresponding author upon reasonable request.

## Conflicts of interest

None.
